# Polyethylene Glycol Functionalized Graphene Oxide Nanoparticles Loaded with *Nigella sativa* Extract: A Smart Antibacterial Therapeutic Drug Delivery System

**DOI:** 10.3390/molecules26113067

**Published:** 2021-05-21

**Authors:** Mustafa A. Jihad, Farah T. M. Noori, Majid S. Jabir, Salim Albukhaty, Faizah A. AlMalki, Amal A. Alyamani

**Affiliations:** 1 Department of Physics, College of Science, University of Baghdad, Baghdad 10071, Iraq; mscmp101@gmail.com (M.A.J.); farah_t258@yahoo.com (F.T.M.N.); 2 Division of Biotechnology, Department of Applied Science, University of Technology, Baghdad 10066, Iraq; 3 Department of Basic Sciences, University of Misan, Maysan 62001, Iraq; 4 Department of Biology, College of Science, Taif University, P.O. Box 11099, Taif 21944, Saudi Arabia; fa.ahmad@tu.edu.sa; 5 Department of Biotechnology, College of Science, Taif University, P.O. Box 11099, Taif 21944, Saudi Arabia; a.yamani@tu.edu.sa

**Keywords:** graphene oxide nanoparticles, polyethylene glycol, *Nigella sativa*, smart drug delivery system, antibacterial activity

## Abstract

Flaky graphene oxide (GO) nanoparticles (NPs) were synthesized using Hummer’s method and then capped with polyethylene glycol (PEG) by an esterification reaction, then loaded with Nigella sativa (*N. sativa*) seed extract. Aiming to investigate their potential use as a smart drug delivery system against Staphylococcus aureus and Escherichia coli, the spectral and structural characteristics of GO-PEG NPs were comprehensively analyzed by XRD, AFM, TEM, FTIR, and UV- Vis. XRD patterns revealed that GO-PEG had different crystalline structures and defects, as well as a higher interlayer spacing. AFM results showed GONPs with the main grain size of 24.41 nm, while GONPs–PEG revealed graphene oxide aggregation with the main grain size of 287.04 nm after loading *N. sativa* seed extract, which was verified by TEM examination. A strong OH bond appeared in FTIR spectra. Furthermore, UV- Vis absorbance peaks at (275, 284, 324, and 327) nm seemed to be correlated with GONPs, GO–PEG, *N. sativa* seed extract, and GO –PEG- *N. sativa* extract. The drug delivery system was observed to destroy the bacteria by permeating the bacterial nucleic acid and cytoplasmic membrane, resulting in the loss of cell wall integrity, nucleic acid damage, and increased cell-wall permeability.

## 1. Introduction

The most promising carbon derivatives in material science are graphene oxide (GO) with excellent physical properties, biocompatibility, and chemical stability [1,2]. In 2004 graphene oxide was first reported as a mono-atomically thick planar layer with two-dimensional (2D) [3]. Variety biomedical and physical applications of GO have been documented by research groups such as drug loading and delivery [4]. A number of hydrophilic oxygen-containing functions in GO such as epoxy, hydroxyl, and carboxyl groups, have improved dispersibility in solvents as well as provide reactive sites for more functionalization by specific interactions which led to the evolution of smart nano carrier-based drug delivery system. This system promises to apply drugs to specific bacterial strains [5,6] and used instead of chemotherapy as anti-cancer drugs [7]. Biocompatible drugs are considered suitable to prevent the immune system from phagocytosing GO in the body. Because of its low absorption by the high aqueous solubility and reticuloendothelial system, polyethylene glycol (PEG) is one of these biocompatible polymers [8]. Mediterranean region herbs *N. sativa* (black seed) is an active herb used as antibacterial therapy. The seeds of *N. sativa* contain 36–38% fixed oils, alkaloids, proteins, and saponin, as well as 0.4–2.5% essential oil. Unsaturated fatty acids make up most of the fixed oil. Thymoquinone cymene, carvacrol, t-anethole, 4-terpineol, and longifolene were among the major components identified by GC-MS, with percentages of (27.8–57.0%), (7.18–15.5%), (5.8–11.6%), (0.25–2.3%), (2.0–6.6%), and (1.0–8.0%), respectively [9,10]. In this work, graphene oxide synthesis by Hummers method and the conjugated of PEG-4000 was done by using EDC hydrochloride C6H17N3–HCl and a catalyst (N–hydroxysuccinimide) for esterification to occur between GO and PEG forming PEGylated graphene oxide (GO-PEG). The *N. Sativa*-loaded GO-PEG (GO-PEG-*N. Sativa*) was obtained by loading the GO-PEG through π-stacking and was then characterized using XRD, FTIR, UV-Vis, Raman, AFM, TEM, and FESEM. Both *E. coli* and *S. aureus* studied their biological activity by examining their effect on the bacteria’s cell-wall morphology, which was observed using SEM. Fluorescence microscopy was used to test the damage that occurred as a result of bacterial DNA using an AO/EtBr staining process. In this study, Flaky graphene oxide (GO)nanoparticles (NPs)were synthesized using Hummer’s method and then capped with polyethylene glycol (PEG) for the first time via an esterification reaction, then loaded with Nigella sativa (*N. sativa*) seed extract. The spectral and structural characteristics of GO-PEG NPs were comprehensively analyzed by XRD, AFM, TEM, FTIR, and UV-Vis to investigate their possible use as a smart drug delivery device against Staphylococcus aureus and Escherichia coli. The drug delivery system was observed to destroy bacteria by permeating the bacterial nucleic acid and cytoplasmic membrane, resulting in the loss of cell wall integrity, nucleic acid damage, and increased cell-wall permeability.

## 2. Material and Methods

### 2.1. Reagents and Chemicals

Natural graphite rod (99.995%), KMnO_2_ (99%), NaNO_3_ (99.5%), H_2_O_2_ (32%), HCl (37.5%), EDC hydrochloride C_6_H_17_N_3_–HCl, N–hydroxysuccinimide (98%) from (Sigma-Aldrich, St. Louis, MO, USA), H_2_SO_4_, (98%) from (LOBA Chemie, Maharashtra, India), NaOH (99%) from Dae-Jung, PEG 4000 from HI Media, Diethylether (C_2_H_5_)_2_O (98%) from Thomas Baker, and ethanol C_5_H_6_OH (99.9%) from Scharlab were used.

### 2.2. Synthesis of Graphene Oxide Nanoparticles (GONPs)

The most economical way used to synthesis GO is Hummer’s method. In which 1g of graphite rod milling in to form a powder and added with sodium nitrate in sulfuric acid was mix in an ice bath for 45 min. After that 3 g of KMnO_2_ was added gradually at 35 °C. 

The thick blend stirred for 1 day. KMnO_2_ is reduced by adding (H_2_O_2_, 32%). A total of 5 mL of (HCl, 37.5%) and DI water used to wash the thick blend. The resultant was dried at 75 °C for 5 h to get the graphene oxide.

### 2.3. Preparations of PEG 4000-Functionalized GO

The activation of (GO-COOH) was begun by the esterification reaction between the GO carboxylic acid group and the PEG hydroxyl group which is necessary for PEGylation of GO, thus, 120 mg/mL of NaOH was dissolved in 20 mL of DI water and added to 20 mL of GO suspension, 2 mg/mL, which was sonicated for an hour and stirred for 3 h. The suspension PH was reduced by adding 3 mL of HCl, then it was centrifuged twice at 4000 rpm for 30 min to produce GO carboxylic acid (GO-COOH). The residue was washed with water twice and the (GO-COOH) was diluted by 10 mL water. To activate (GO-COOH), 400 mg of EDC and 240 mg of NHS were added to (GO-COOH), then for (GO-PEG), 1500 mg of PEG4000 was added to the resultant and stirred for 24 h. In the final step, the resultant was centrifuged and washed by DI water. The results of carboxylation and PEGylation conjugation were characterized using FTIR and confirmed using UV-vis. 

### 2.4. Loading Nigella sativa (N. sativa) on GONPs-PEG

After ethanol extraction of *N. sativa* seed, 2.250 mL of *N. sativa* oil (3.33 mg/mL) was loaded on 7 mL aqueous suspension (4.28 mg/mL) GO-PEG. The *N. sativa* oil and GO-PEG were stirred for 24 h at a 1:4 ratio of *N. sativa* oil to GO-PEG. The suspension was centrifuged (10,000 rpm, 25 °C) for 60 min. and left in the oven for 12 h at 40 °C to dry and produce the final sample, which is called GO-PEG-*N. sativa*.

(1) $$druge\;loading\;content\left(\%\right))=\frac{weight\;of\;druge\;in\;nanoparticles}{weight\;of\;nanoparticles}\times 100$$

### 2.5. Antibacterial Activity of GONPs-PEG-N. sativa

The antibacterial ability of the processed GO-PEG was examined using an agar well diffusion technique against the two bacterial strains *E.* *coli* (Gram-negative) and *S. aureus* (Gram-positive) [11,12]. Before starting the culturing process, 20 mL of Muller-Hinton (M-H) was poured to the Petri dishes. A sterile wire loop was used to capture the bacteria from their stock cultures. Following the culturing of the cells, a sterile tip was used to bore six mm-diameter wells in the agar plates [13,14]. The different concentrations of the GONPs, GONPs-PEG, and GONPs-PEG- *N. sativa* (62.5,125, 250, 500 μg/mL) were added to the boring plates. After culturing, the plates were incubated at 37 °C overnight. The tests were done in triplicate [15]. To assess how GONPs-PEG- *N. sativa* affects the growth curve of bacteria, they were cultured on the M-H agar plates at 37 °C, the collection freshly cultured plates inoculations composed of 50 mL of nutrient broth. The bacterial grew until the nutrient broth reached optical density (OD) of 0.1 at 600 nm, which is equivalent to the bacterial concentration of corresponds a bacterial concentration of 10^8^ (CFU/mL). Then, bacterial cultures (1 mL) were added to the nutrient broth and were supplemented with GONPs, GONPs-PEG, and GONPs-PEG-*N. sativa* at the 250 μg/mL concentration and incubated at 37 °C for 12 h with slight agitation. A spectrophotometer was used to determine bacterial growth by measuring the OD [16]. 

### 2.6. Morphology of Bacterial Strain Using SEM

The divers in the morphology of the two bacterial strains *E. coli* and *S. aureus* were observed using a scanning microscope. Both treated and untreated bacteria cells were centrifuged at 500 rpm and then washed three times using 50 mM, phosphate buffer solutions (pH 7.3). A thin suspension film was made on a clean silicon wafer slide. They were then air-dried at ambient temperature and then they were fixed using 1 mL of a fixing buffer. Once they were fixed, the slides were incubated at 37 °C for 1–2 h, and the water was removed using methanol (ascending grade), dried in the open air, and then fixed on the SEM stubs, and they were coated with gold for 5 min, about 20 nm of gold was left on the cells surface. SEM (TESCAN, Vega III, Czech Republic) which is a multifunctional instrument that can be used to obtain images of a material’s surface structure and morphology was used to examine the gold-coated cells [17]. For TEM analysis, a transmission electron microscope (JEOL Ltd., Tokyo, Japan) with an operating voltage of 80 kV was used. In order to conduct TEM examination, 5 L drops of aqueous suspensions containing PEG-coated GO NPs were put on carbon-coated copper grids. After 15 min, the excess water was collected by the filter paper, and the samples were left to dry in the open air while TEM images were taken.

### 2.7. Detection of Reactive Oxygen Species (ROS)

The ROS that was released by both treated and untreated bacterial cells were detected using an Acridine orange-ethidium bromide (AO/EtBr) staining procedure. A fluorescent microscope was used to measure the antibacterial activity of the GONPs, GO-PEG, and GONPs-PEG-*N. sativa* on the organisms for the study. To distinguish cell viability after treatments an AO/EtBr staining procedure was done. A total of 50 μL bacterial suspension of both treated and untreated was mixed with 50 μL (prepared from 10 μg/mL AO/EtBr stock solution) and was left for about 2 min. The staining procedure followed, after which a thin film of the mixture was applied on a glass slide and then observed under an immunofluorescent microscope. For the living cells, the Acridine orange-stained fluoresce green while for the dead cells the ethidium bromide-stained fluorescence red [18].

### 2.8. Bacterial Adherence Assay

The cells of the rat embryonic fibroblast were cultured in twelve well tissue culture plates at 1 × 10^5^. These cells were contacted with bacteria strains at a multiplicity of infection (MOI) ratio of 200:1 under the absence and presence of the GONPs, GONPs-PEG, and GONPs-PEG-*N. sativa* at a concentration of 100 μg/mL. The plates were then incubated for 1 h under a 5% CO_2_ incubator at 37 °C. After incubation, they were washed thrice with PBS. PFA was used to fix the cells for 15 min, and later crystal violet stain was used to stain the cells for 15 min.

### 2.9. Bacterial Invasion Assay

A 12 well tissue was also used to grow rat embryonic fibroblast (REF) cells, which were later infected with bacterial strains in the absence and presence of GONPs, GONPs-PEG, and GONPs-PEG-*N. sativa* at concentration 100 μg/mL for 2 h. The REF cells left after culture media was removed were washed thrice with PBS. A fresh RPMI-1640 medium consisting of gentamicin 100 μg/mL was added and the mixture incubated for 2 h. The cells were then washed thrice with PBS, broken down for 20 min using 0.1% Triton X-100 at 37 °C. For each well, 10 μL was drawn and added to the nutrient agar for bacteria colonies to grow, which were counted after 20 h. The bacteria invasion efficiency was determined as the mean number of bacterial in each well. This bacterial invasion assay was conducted in triplicate [19]. 

### 2.10. Statistical Analysis

The unpaired t-test was the technique used to analyze our results as allows comparison of experimental groups at a significant *p*-value of <0.05 [20].

## 3. Results and Discussion

### 3.1. Structural Properties of GO and GO-PEG

**Figure 1:**
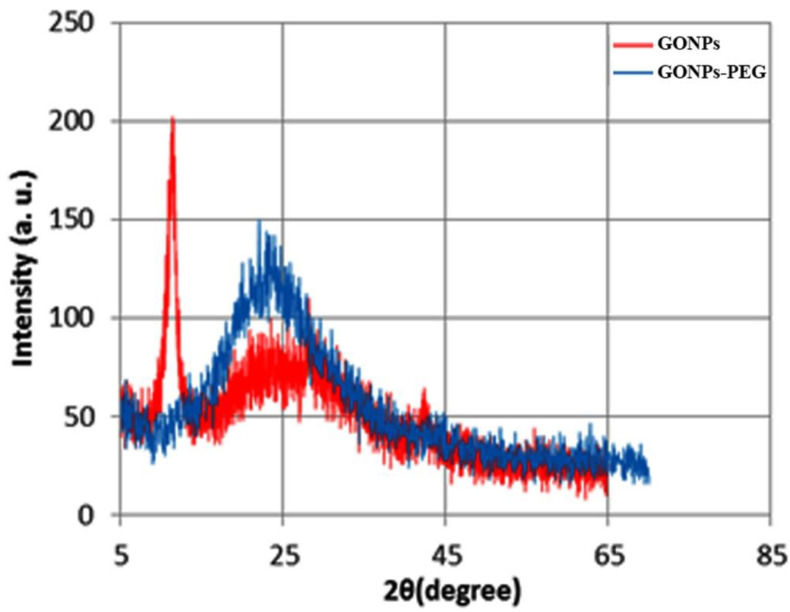
XRD patterns of GONPs and GONPs–PEG.

Figure 1 shows XRD patterns for GONPs and GONPs-PEG in the red line. GONPs were showed a sharp peak that appeared at 2θ = 11.83° that related to (001) plane with d–spacing (7.76°) and conform the hexagonal structure of GONPs. The blue line showed the functionalization of GONPs–PEG 4000 with a broad peak at 2θ = 23.38° with d–spacing (3.8 Å) which is results from functionalized while the peak of GONPs disappear because GONPs has an effect on the PEG molecular chain structure in the crystal lattice, disrupting the order of its crystallization. This reduces the crystallinity of PEG and results in an effective PEG to GO Nano-sheets by ester bonding [21]. The indexing of (001) determines by the high score plus program.

### 3.2. Morphological Properties of GO, GO–PEG and GO-PEG-N. sativa

**Figure 2:**
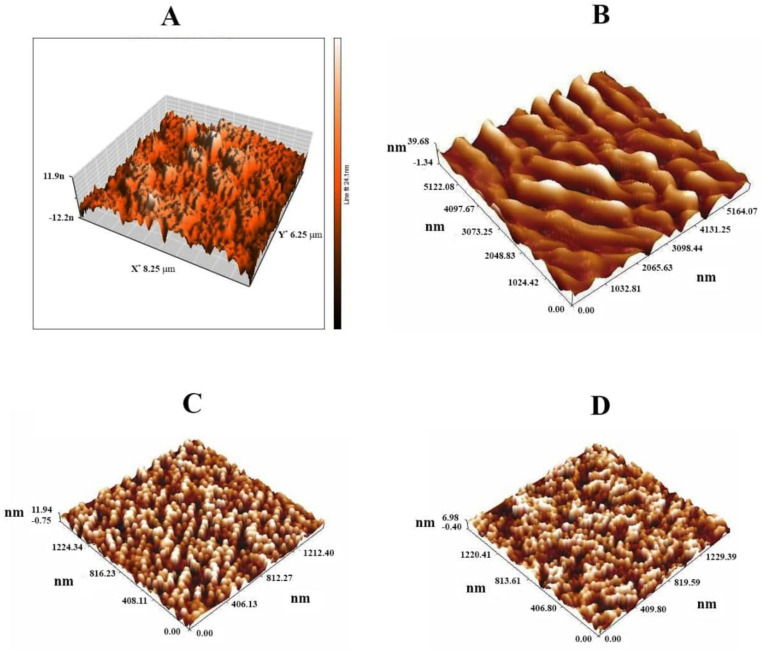
AFM images of (**A**): GONPs, (**B**): GONPs-PEG, (**C**): *N. sativa* and (**D**): GONPs-PEG-*N. sativa*.

**Figure 3:**
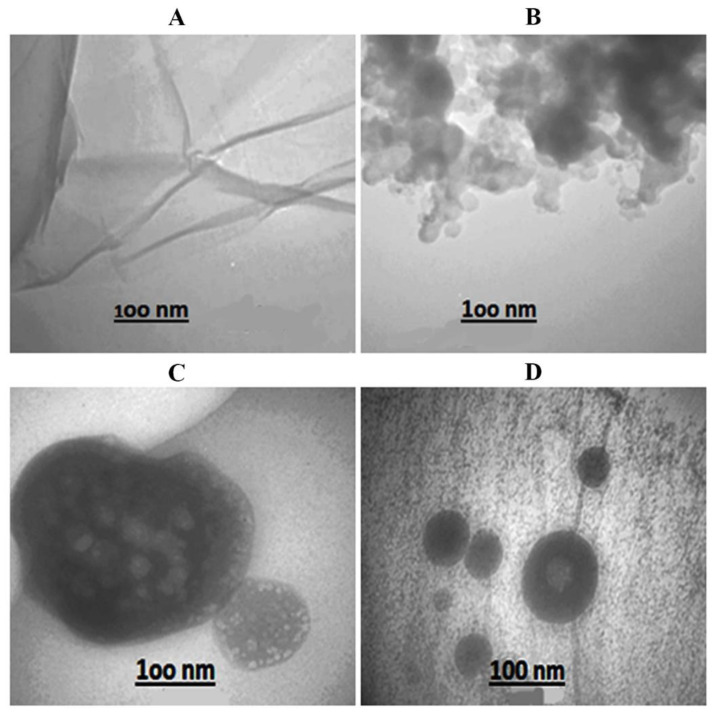
TEM images of (**A**): GONPs, (**B**): GONPs-PEG, (**C**): *N. sativa* and (**D**): GONPs-PEG-*N. sativa*.

AFM and TEM were used to examine the morphology and size of GONPs, GONPs–PEG, and GONPs–PEG–*N. sativa*, as shown in Figure 2 and Figure 3. Images of GONPs showed graphene sheets with the main grain size of 24.41 nm which conform to the nanosize of synthesis while images of GONPs-PEG illustrated aggregation of graphene oxide with PEG4000. The main grain size increase with functionalizing to 287.04 nm. As a result of the extraction *N. sativa* seed the main grain size is 257 nm and it was increased when adding the *N. sativa* to GONPs-PEG to reach 295 nm. The spherical shape is a domain in the last images with conjugation of extraction seed with polymer, and it was showed a significant difference between GONPs, GONPs-PEG, GONPs-PEG-*N. sativa*. The GONPs sheets images obtained by TEM were found to be single-layered and wrinkled. The particle size was recorded to less than 100 nm as viewed in Figure 3. The conjugation of PEG to GONPs led to an increase, more than 100 nm, in the size of the nanomaterial. The *N. sativa* seed extraction showed a spherical shape with a size of more than 100 nm while the designed drug of GONPs-PEG-*N. sativa* showed tree-like of conjugated polymer with *N. sativa*. 

### 3.3. Chemical Properties of GO-PEG-N. sativa

**Figure 4:**
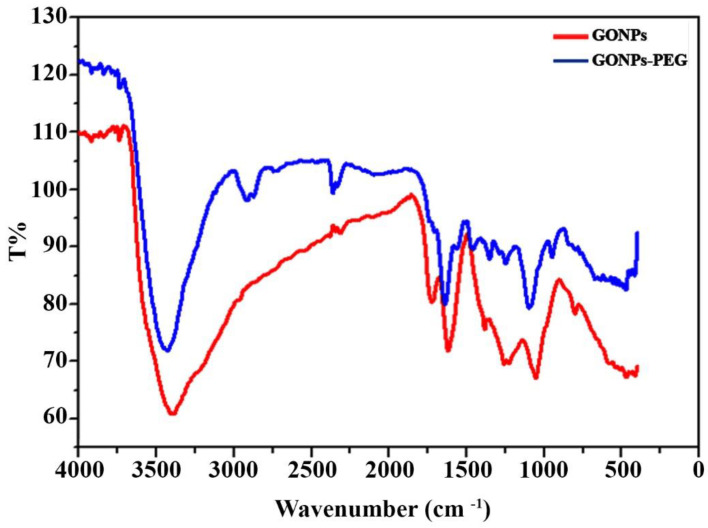
TIR spectrum of GONPs and GONPs-PEG.

**Figure 5:**
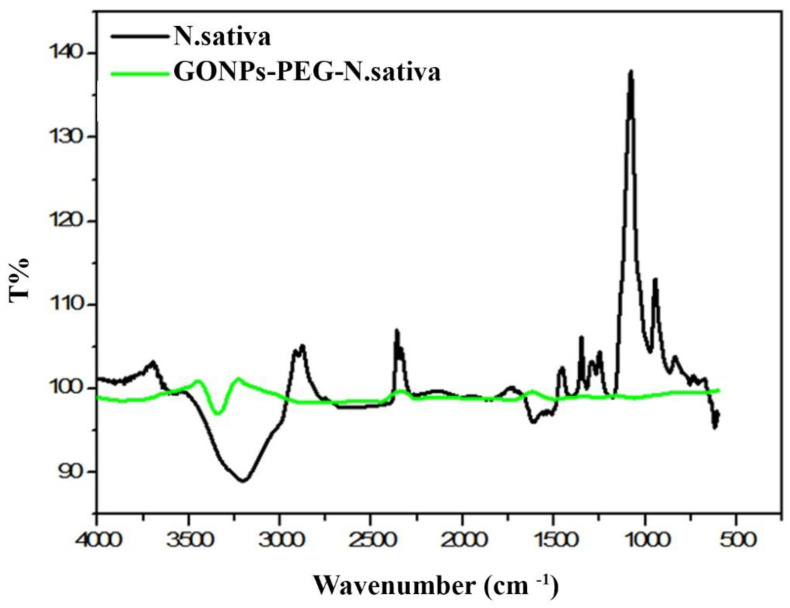
FTIR spectrum of *N. sativa* extract and GONPs-PEG-*N. sativa*.

FTIR spectroscopy was used to detect changes in the characteristic bands of GONPs and after GONPs–PEG coupling. In general, amino acids are zwitterions that have spectra that contain both primary amine and carboxylate functional groups [22]. Figure 4 shows the FTIR spectrum of GONPs; the peaks at 3338 cm^−1^ are stretching related to OH, while 1618, 1382, and 1026 cm^−1^ are C=O groups in carbonyl [-C(=O)-] and carboxyl (-COOH) and these results are consistent with a previous study published by Charmi J et al. [23]. The peak at 1724 cm^−1^ is related to C=C and peaks at 1066 and 1382 are related to C-O [24]. In the case of GONPs–PEG, the peak at 3338 cm^−1^ was shifted to 3427 cm^−1^ and the appearance of C-H at 3920 cm^−1^ proved the conjunction of GONPs to PEG. C=C, C-O, and C=O were found at 1641, 1390, and 1107 cm^−1^, respectively [25]. The FTIR pattern of *N. sativa* extract was shown in Figure 5, which shows a broad and strong band at approximately 3209 cm^−1^, which represented the stretching vibration of O-H in the hydroxyl groups of hydrogen bonds [26]. The region between 1771 cm^−1^ and 621 cm^−1^ is may be assigned to C-O stretching vibrations. The small bands at 578, 563, and 621cm^−1^ were attributed to the out-of-plane bending vibrations of C-H in the benzene derivative. The Greenline illustrates the loading drug GONPs-PEG-*N. sativa*, and the OH stretching of GO–PEG is shifted to 3745 cm^−1^ while the peaks 2854–2924 cm^−1^ C-H stretch (Alkane), which is also appeared at 1516 cm^−1^. The region between 756 cm^−1^ and 439 cm^−1^ is may be assigned to C-O stretching vibrations (ester) [27].

### 3.4. Optical Properties of GO-PEG-N. sativa

**Figure 6:**
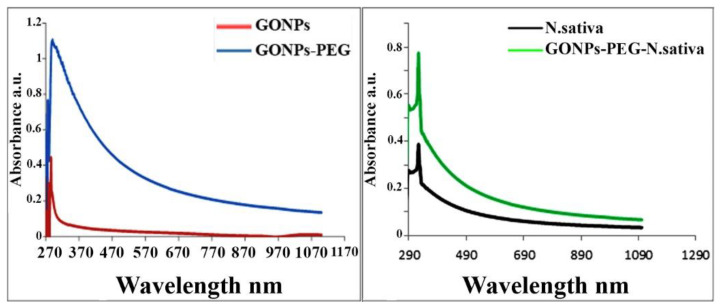
UV-vis absorption spectrum of GONPs, GONPs-PEG, *N. sativa*, GONPs-PEG-*N. sativa*.

In UV-vis spectra of GONPs and GONPs–PEG (Figure 6), the red line indicates the GONPs spectra at 275 nm which represent the absorption bands that related to electronic transition π–π* for C-C (aromatic rings) and transition n–π* for C=O in the GO. The blue line represents the GONPs–PEG that shifted to 284 nm which is confirmed the synthesis of GONPs–PEG [28]. The black line illustrates the spectra of *N. sativa* and the green line represented GONPs-PEG-*N. sativa*. In *N. sativa* showed a sharp peak at 324 nm while in GONPs-PEG-*N. sativa* the peak shifted to 327 nm. 

### 3.5. Antibacterial Activity of GO, GO–PEG, GO–PEG–N. sativa

The antibacterial effects of pure gold nanoparticles are viewed differently by researchers. Many researchers conclude that pure nanogold particles have no antibacterial properties or that the results are minimal. According to a study conducted by Zhang et al., gold nanoparticles have no or only slight bactericidal effects at high concentrations [29]. The effect of 15 nm gold nanoparticles on the antibacterial activity of gentamicin was investigated by Burygin et al. using a variety of methods at various concentrations of gentamicin and particles, but no differences in antibacterial activity of gentamicin and gentamicin-gold nanoparticle mixtures were found within the range of experimental errors. [30].

**Figure 7:**
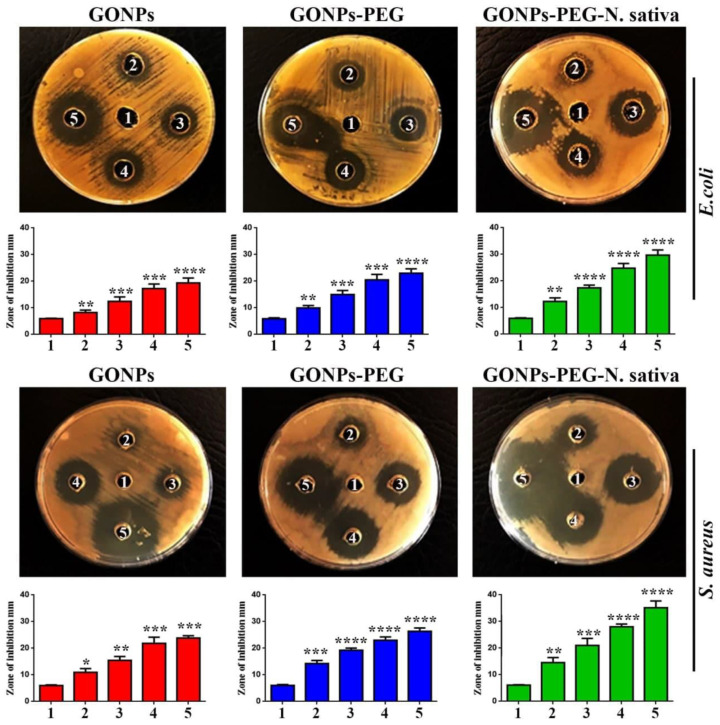
Anti-bacterial activity of GONPs, GONPs–PEG, and GONPs–PEG–*N. sativa* against *S. aureus* and *E. coli.* (1): control untreated bacterial strains. The inhibited zones of bacterial growth for both strains are illustrated when exposed to variety concentrations as follows; (2): 62.25 µg/mL, (3): 125 µg/mL, (4): 250 µg/mL, (5): 500 µg/mL. The data are shown as the mean ± SD. * *p* < 0.05, ** *p <* 0.01, *** *p <* 0.001, and **** *p <* 0.0001.

**Figure 8:**
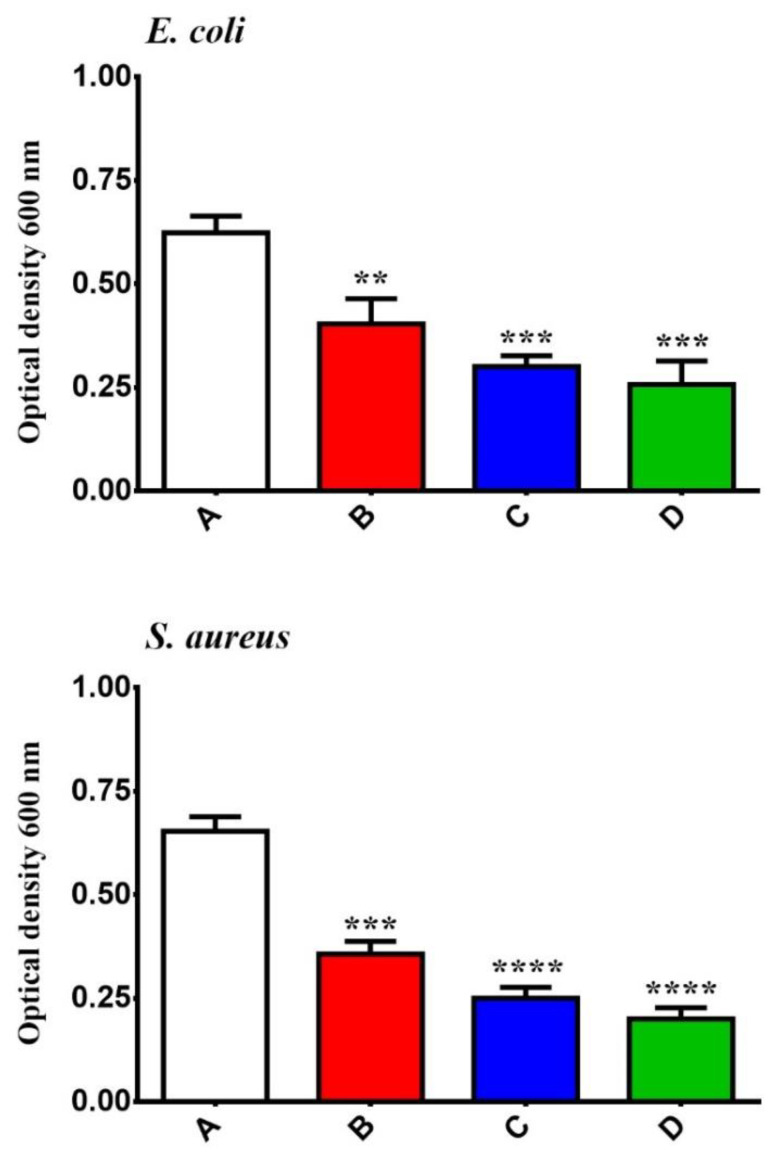
Effect of GONPs, GONPs–PEG and GONPs–PEG–*N. stiva* in the growth rate of *S. aureus* and *E. coli*. cell (**A**), bacterial cells treated with GONPs (**B**), with GONPs–PEG (**C**), and with GONPs–PEG–*N. sativa* (**D**). The data are shown as the mean ± SD. ** *p <* 0.01, *** *p <* 0.001, and **** *p <* 0.0001.

On the other hand, several studies have shown that Metal Oxide NPs have antibacterial properties against *S. aureus* and *E. coli* [31,32]. The antibacterial of GONPs, GONPs–PEG, GONPs– PEG–*N. sativa* was analyzed using *S. aureus* and *E. coli.* The inhibition zone after organism’s exposure to different concentrations of GONPs, GONPs–PEG, GONPs–PEG–*N. sativa* were measured and illustrated in Figure 7. The result showed that the GONPs–PEG–*N. sativa* was found to be effective more than GONPs and GONPs–PEG and the results are concentration-dependent manner. GONPs–PEG–*N*. *sativa* produced a zone of inhibition with a diameter approximately 30 mm against *E. coli* and 36 mm against *S. aureus*. A zone of inhibition was produced at the concentration (500 μg/mL) with a diameter of almost 36 mm against *S. aureus* and a diameter of 30 mm against *E. coli*. The results demonstrated that the effect of the nanoparticles depended on the concentration. The micro-organisms demonstrated resistance to external agents because their outer membrane has a bacterial structure [33]. The GONPs, GONPs–PEG, GONPs–PEG–*N. sativa* effect on the growth of the organisms was time-dependent, with more growth noticeable after 12 h. of treatment as shown in Figure 8. Taken together, the inhibitory effect of GONPs–PEG–*N. sativa* was observed to be more than of GONPs, GONPs–PEG as proven by the statistical analysis.

### 3.6. Bacterial Morphology

**Figure 9:**
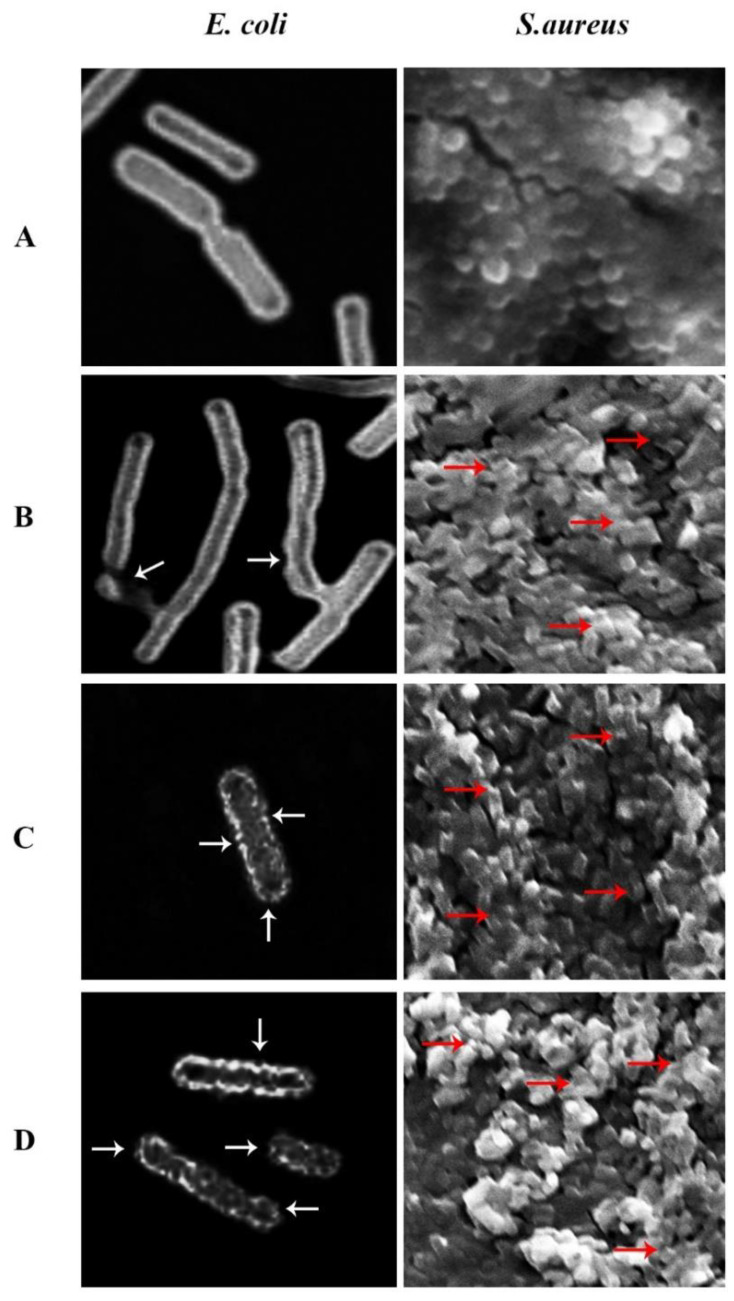
SEM images visualized the effect of GONPs, GONPs–PEG, and GONPs–PEG–*N. sstiva* on treated *S. aureus* and *E. coli*. The bacterial strains showed changes in the cell membranes like damaged, blabbed, and clumped membranes. Non-treated bacterial cell (**A**), bacterial cells treated with GONPs (**B**), with GONPs–PEG (**C**), and with GONPs–PEG–*N. sativa* (**D**).

The effect of GONPs, GONPs–PEG, GONPs–PEG–*N. sativa* on the structure of organisms under treatment was assessed using the SEM technique. The images demonstrated that there were differences in the bacteria cell morphology between treated samples GONPs, GONPs-PEG, GONPs–PEG–*N. sativa* and the untreated samples (control). Untreated control bacterial strain *E. coli* SEM images confirmed the presence of cells as rod-form colonies as in Figure 9A. Since *S. aureus* is Gram-positive bacteria, and thus, exists in clusters, SEM images demonstrated that they were destroyed after they were treated with GONPs, GONPs–PEG, GONPs–PEG–*N. sativa* and as shown in Figure 9. The GONPs were observed to have moderate activities on bacterial strains as demonstrated in the bacterial cell structural changes in Figure 9B. The GONPs-PEG had more effect on the tested microorganisms’ outer membrane, as it was observed that the bacterial strain cell membrane had more pores when it was treated with GONPs–PEG. The damage occurred as a result of osmotic imbalance leading to a leak of bacterial cells and it resulted in changes in morphology, osmotic balance, and cells’ structural integrity after it was treated with GONPs-PEG as in Figure 9C. Furthermore, GONPs-PEG–*N. sativa* had higher damage in bacterial strains as shown in Figure 9D. It was observed that in the bacterial strains treated with GONPs, GONPs–PEG, GONPs–PEG–*N. sativa* there was aggregation and membrane rupture compared to the untreated strains. 

### 3.7. GONPs, GONPs–PEG and GONPs–PEG–N. sativa Induces Production of ROS

**Figure 10:**
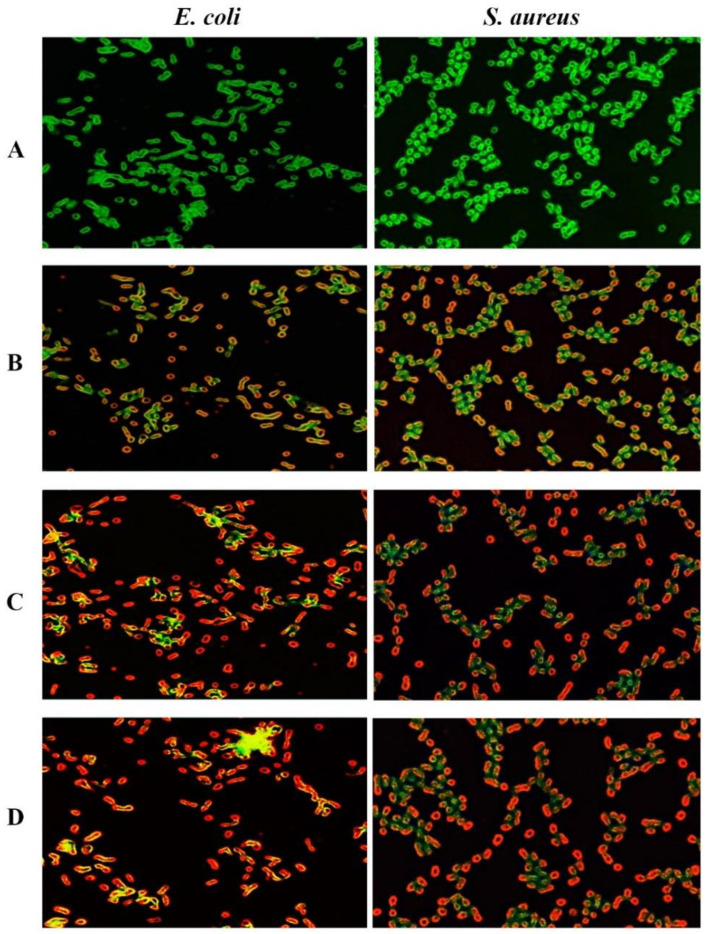
Fluorescence microscopic images of the green and red fluorescence stained *S. aureus* and *E. coli*. (**A**): non-treated bacterial strains. Bacterial cells treated with; (**B**) GONPs, (**C**): GONPs–PEG and (**D**): GONPs–PEG–*N. sativa*.

The AO/EtBr staining technique was used to detect the presence of ROS after the bacterial strains were treated with GONPs, GONPs–PEG, GONPs–PEG–*N. sativa*. The indicators that show the presence of ROS are nitric oxide and hydrogen peroxide. When AO/EtBr dye comes into contact with reactive oxygen species, produced when an organism is under stress, it undergoes oxidation. The EtBr component will only pervade cells whose membrane integrity has been damaged and reacts with cells nucleic acid. The dead cells are stained in red while the viable cells are stained green [34,35]. The bacterial strains that were treated with GONPs, GONPs–PEG demonstrated moderate malformations compared to the untreated cells as present in Figure 10B,C. The GONPs–PEG–*N. sativa* treated bacterial stains resulted in more structural deformities as well as higher levels of ROS production as in Figure 10D, as demonstrated by a high number of bacteria strains that are reddish. Overall, the results showed that GONPs, GONPs–PEG, GONPs–PEG–*N. sativa* were suitable as antibacterial agents that can be applied in biomedical and biological fields. 

### 3.8. GONPs, GONPs–PEG and GONPs–PEG–N. sativa Attenuated Invasion of Bacterial Strains to REF Cells

**Figure 11:**
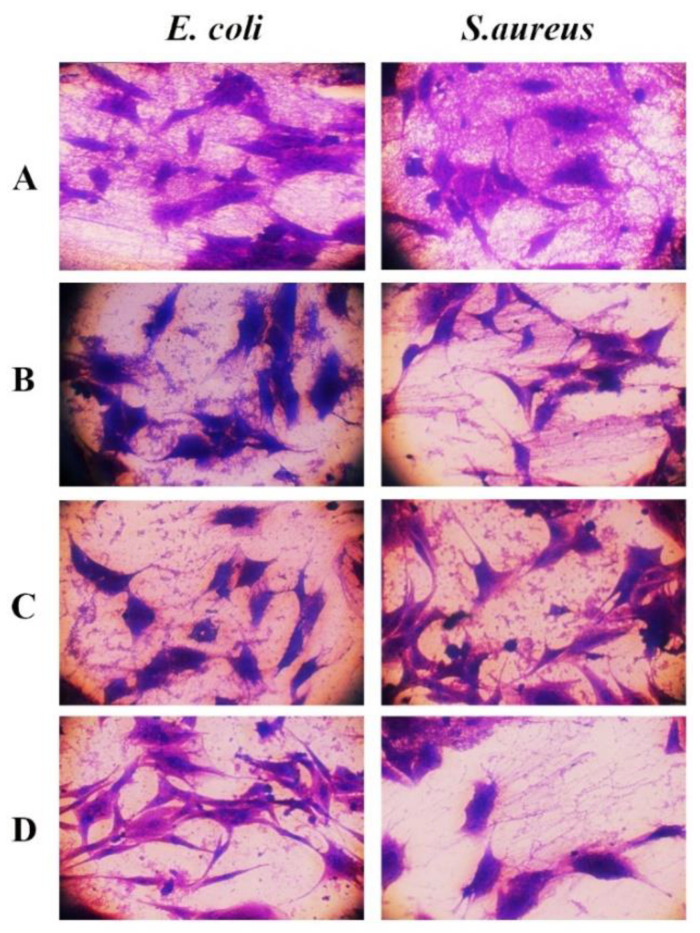
GONPs, GONPs–PEG and GONPs–PEG–*N. sstiva* inhibits invasion of bacterial strains in REF cells as indicated; (**A**): control REF cells infected with bacterial strains, (**B**): REF cells pre-treated with GONPs then infected with bacterial strains, (**C**): REF cells pre-treated with GONPs-PEG then infected with bacterial strains, (**D**): REF cells pre-treated with GONPs–PEG-*N. sativa* then infected with bacterial strains.

**Figure 12:**
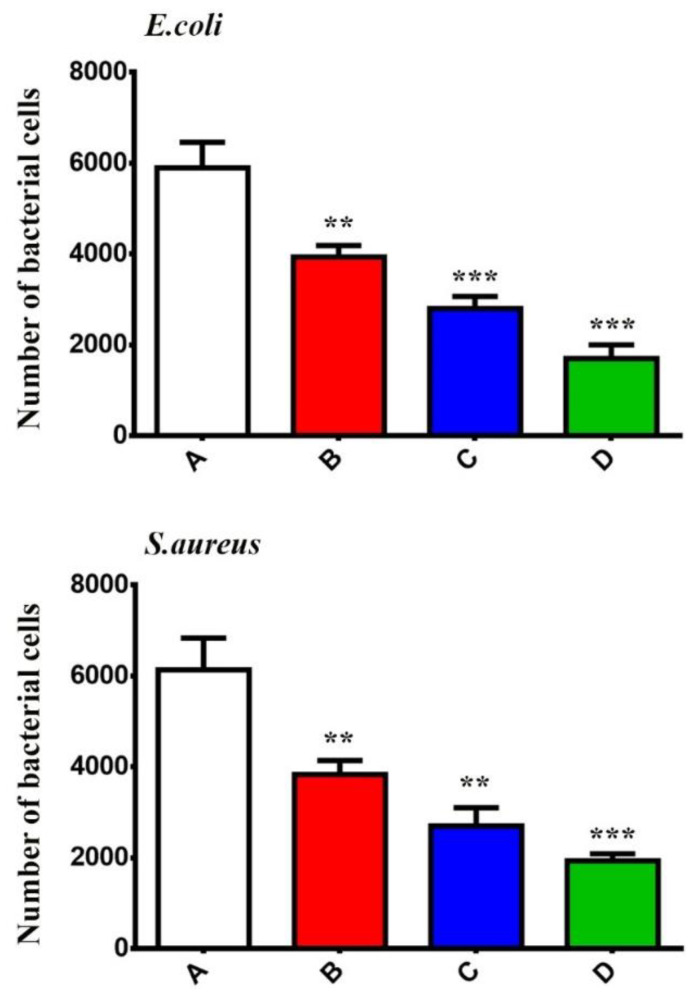
Illustrates the decrease interaction of bacterial strains with REF cells pre-treated with GONPs, GONPs–PEG, and GONPs–PEG–*N. sativa*. (**A**): control REF cells infected with bacterial strains, (**B**): REF cells pre-treated with GONPs then infected with bacterial strains, (**C**): REF cells pre-treated with GONPs-PEG then infected with bacterial strains, (**D**): REF cells pre-treated with GONPs –PEG-*N. sativa* then infected with bacterial strains. The values are shown as the mean ± SEM. ** *p <* 0.005, *** *p <* 0.001.

REF cells underwent pretreatment with GONPs, GONPs–PEG, GONPs–PEG–*N. sativa* for an hour at a concentration of 100 μg/mL and then the bacterial strains were used to infect the cells at MOI (200:1). It was observed that the GONPs, GONPs–PEG, GONPs–PEG–*N. sativa* resulted in the attenuation of bacterial strains binding to REF cells as shown in Figure 11. To determine whether GONPs, GONPs–PEG, GONPs–PEG–*N. sativa* also can hinder bacterial strains invasion, REF cells were pretreated GONPs, GONPs–PEG, GONPs–PEG–*N. sativa* at a concentration of 100 μg/mL for an hour and then they were infected with bacterial strains for three hours. It was observed that there was a significant decrease of cell invasion of bacterial strains in the presence and absence of GONPs, GONPs–PEG, GONPs–PEG–*N. sativa* as shown in Figure 12. Taken together, these results of the present study demonstrated that the GONPs, GONPs–PEG, GONPs–PEG–*N. sativa* plays a mediation role in the invasion and adherence of bacterial strain in REF cells, and these findings are consistent with previous studies [36].

## 4. Conclusions

In conclusion, flaky single-layer graphene oxide (GO) synthesis by Hummer’s method using rod graphite, polyethylene glycol (PEG4000) was capped with GONPs. This reaction happened between the graphene oxide (GO) carboxylic acid group and the polyethylene glycol (PEG) hydroxyl group to active (GO-COOH) using organic material and the results are GONPs–PEG smart drug delivery. The drug-loaded by *Nigella sativa* to get GONPs-PEG-*N. sativa*. This study investigated the antibacterial activity of functionalized graphene oxide with PEG4000 loading *Nigella sativa*. From the results the loading drug GONPs- PEG-*N. sativa* showed inhibitory more than GONPs and functionalized GONPs-PEG. The nanoparticles exhibited more activity in *S. aureus* as compared to the *E. coli*. Our study demonstrated that the preparation and characterization of the GONPs, functionalized nanoparticles GONP–PEG, and drug GONPs-PEG-*N. sativa* was successful and demonstrated a potential antibacterial activity against both *S. aureus* and *E. coli*.

## Data Availability

Not applicable.

## Abbreviations

GONP	graphene oxide nanoparticles
GONPs–PEG	graphene oxide nanoparticles–polyethylene glycol
